# A comparison of commercially-available automated and manual extraction kits for the isolation of total RNA from small tissue samples

**DOI:** 10.1186/s12896-014-0094-8

**Published:** 2014-11-14

**Authors:** Marlo K Sellin Jeffries, Andor J Kiss, Austin W Smith, James T Oris

**Affiliations:** Department of Biology, Texas Christian University, Fort Worth, TX 76129 USA; Department of Biology, Miami University, Oxford, OH 45056 USA; Center for Bioinformatics and Functional Genomics, Miami University, Oxford, OH 45056 USA

**Keywords:** RNA extraction, Gene expression, qPCR, RNA-Seq, Next generation sequencing, Microarray, Small tissues, Fathead minnow

## Abstract

**Background:**

This study compared the performance of five commercially available kits in extracting total RNA from small eukaryotic tissue samples (<15 mg). Total RNA was isolated from fathead minnow (*Pimephales promelas*) tissues (spleen, blood, kidney, embryo, and larvae) using the Qiagen RNeasy® Plus Mini, Qiagen RNeasy® Plus Universal, Promega Maxwell® 16 LEV simplyRNA, Ambion MagMAX™-96 and Promega SimplyRNA HT kits. Kit performance was evaluated via measures of RNA quantity (*e.g.*, total RNA amount) and quality (*e.g.,* ratio of absorbance at 260 and 280 nm, RNA integrity number (RIN), presence of gDNA).

**Results:**

With the exception of embryos, each kit generally extracted ≥5 μg of total RNA from each sample. With regard to RNA quality, the RINs of RNA samples isolated via the Plus Mini and Maxwell® 16 kits were consistently higher than those of samples extracted via the remaining three kits and for all tissues, these kits produced intact RNA with average RIN values ≥7. The Plus Universal and SimplyRNA HT kits produced moderately degraded (RIN values <7, but ≥5), while the RNA recovered via the MagMAX™ kit tended to exhibit a high degree of degradation (RIN values <5).

**Conclusions:**

Each kit was generally capable of extracting the amount of RNA required for most downstream gene expression applications suggesting that RNA yield is unlikely to be a limiting factor for any of the kits evaluated. However, differences in the quality of RNA extracted via each of the kits indicate that these kits may differ in their ability to yield RNA acceptable for some applications. Overall, the findings of this study demonstrate that there are practical differences between commercially available RNA extraction kits that should be taken into account when selecting extraction methods to be used for isolating RNA designated for gene expression analysis.

**Electronic supplementary material:**

The online version of this article (doi:10.1186/s12896-014-0094-8) contains supplementary material, which is available to authorized users.

## Background

Gene expression analysis is a well-established method by which changes in the physiological status of cells and tissues can be detected. Previous studies have shown that extraction of high-quality RNA from tissue samples is a key step in the collection of accurate and reliable gene expression data via real-time quantitative PCR (qPCR), hybridization microarrays and next generation sequencing (NGS) technologies (*i.e.*, RNA-Seq) [1-6]. Recovery of sufficient amounts of high-quality RNA from small (<15 mg) and/or complex eukaryotic tissue samples has historically been considered problematic [7-9]. Several manufacturers have recently begun to market kits designed to recover high-quality RNA from these types of samples [10]; however, few studies have directly compared the performance of commercially available kits in isolating RNA from small tissue samples [11].

Although there are differences in the technologies that commercially-available kits employ to isolate total RNA from tissue samples, each uses the same general approach: 1) cell lysis and inhibition of ribonuclease (RNase) activity, 2) removal of DNA, and 3) isolation of total RNA (of which 1-5% is mRNA). Most kits require that tissues be homogenized in a buffer containing detergents and an RNase denaturing agent to lyse cells and inhibit RNase activity. Removal of genomic DNA (gDNA) most often involves the treatment of samples with DNase I enzyme; however, some kits utilize non-enzymatic methods, including liquid-liquid (*e.g.*, gDNA eliminator solution) extractions and solid-phase extractions (*e.g.*, gDNA eliminator spin columns). Several methods are available for the isolation of RNA [12]; those most commonly employed by commercially-available kits include liquid-liquid extraction (*e.g.*, phenol-chloroform, TRIzol, etc.) or solid-phase extraction using silica-based membrane or paramagnetic bead technologies. Though many RNA isolation kits require manual operation, kits designed for use with liquid-handling robots are becoming more readily available due to an increased demand for high-throughput assays.

Regardless of the procedure used for RNA isolation, it is necessary to determine the quantity of the recovered RNA prior to downstream gene expression analysis [13]. Quantitation of RNA can be done via spectrophotometry (UV) or fluorometry (RNA specific dye). The amount of RNA required for downstream analysis depends upon the method utilized for quantifying gene expression. For example, the total RNA amount required for first-strand cDNA synthesis is in the picogram (pg) to nanogram (ng) range [14], while the amount required for microarray analysis is often in the microgram (μg) range [15].

In selecting an RNA isolation kit, not only must the ability of the kit to recover the required amount of total RNA be taken into consideration, but also the ability of the kit to recover RNA meeting specific quality criteria. Total RNA samples to be used in gene expression analysis should be: 1) free of proteins and contaminants that can inhibit downstream molecular analyses, 2) intact and free of nucleases (that can lead to subsequent degradation) and 3) free from gDNA contamination [16]. The degree of sample contamination by proteins and other contaminants is typically evaluated by determining ratios of sample absorbance at 260 and 280 nm (A_260_:A_280_). This ratio estimates the degree of contamination resulting from protein and other contaminants (nucleic acids absorb at 260 nm, while proteins and other contaminants of concern absorb at 280 nm) and total RNA samples with values ≥1.8 are considered “pure” and acceptable for downstream molecular analyses [17]. More recently, a so-called “RNA integrity number (RIN)” or “RNA Quality Indicator (RQI)” has become the benchmark standard for RNA quality assessment. These metrics indicate the degree of RNA fragmentation (with increasing values representing more intact RNA) [18] and can be obtained via lab-on-chip gel electrophoresis systems, derived from LapChip® microfluidics technology (Caliper Life Sciences, Inc.) and marketed by Agilent Technologies (Bioanalyzer2100) and BioRad (Experion). RNA with RIN or RQI values ≥7-8 are typically considered an optimal template for most downstream molecular applications [1-3,7,19,20]; however, the minimum acceptable RIN value depends on the type of analysis to be done. For example, several studies have found RNA with RIN values ≥5-5.5 to provide a suitable template for qPCR reactions in cases where the amplicon length does not exceed 200 bp [1-3,7]. With regard to the evaluation of gDNA contamination, the standard approach involves conducting qPCR reactions on “no-RT” or RT(−) samples [13]. RT(−) samples are not subject to reverse transcription and should thus, contain only RNA, which cannot be amplified in qPCR reactions. Therefore, the detection of a fluorescent signal in RT(−) samples indicates contamination by gDNA.

The goal of this study was to compare the performance of several commercially available manual and automated RNA extraction kits to isolate total RNA from small amounts of vertebrate tissue samples (average mass of <20 mg). To accomplish this goal, total RNA was isolated from fathead minnow (*Pimephales promelas*) tissues (blood, spleens, kidneys, embryos and larvae) using the following kits: 1) RNeasy® Plus Mini Kit (Qiagen, Valencia, CA), 2) RNeasy® Plus Universal Kit (Qiagen), 3) Maxwell® 16 LEV simplyRNA Purification Kit (Promega, Madison, WI), 4) MagMAX™-96 Total RNA Isolation Kit (Ambion/Life Technologies, Grand Island, NY) and 5) SimplyRNA HT System (Promega, Beta test kit). The quantity and quality of RNA extracted from each of these kits was compared in an effort to determine which kits were most appropriate for low- and high-throughput RNA extractions of small tissues.

## Methods

### General experimental design

**Figure 1:**
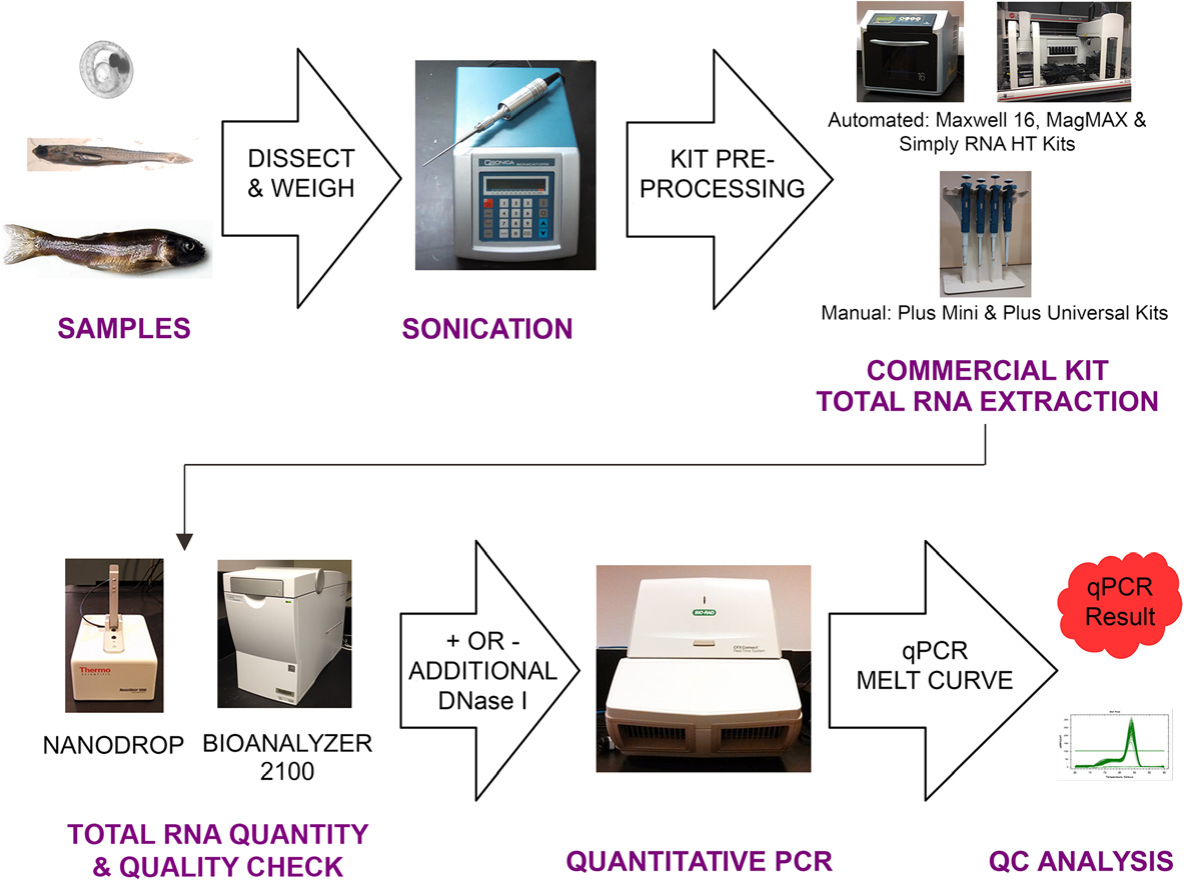
**Visual description of the experimental design utilized to evaluate the performance of the Plus Mini, Plus Universal, Maxwell®, MagMAXTM and SimplyRNA HT RNA extraction kits.**

Figure 1 shows an overview of the experimental design employed in this study. Briefly, blood, kidneys, spleens were obtained from sexually-mature (~7 months old) male fathead minnows. Embryos (~72-84 h post fertilization) and whole larvae (~168 h post hatch) were also collected. A total of eighty samples were randomly assigned for RNA isolation by each of the commercially available kits so that four samples of each tissue type were extracted by each kit. A description of each of the selected kits including the method by which each isolates RNA appears in Table 1. Following the isolation procedures, the quantity and quality of RNA in each sample was evaluated.

**Table 1:** **Descriptions of the five commercial kits selected for inclusion in this study**

**Kit name (Catalog #)**	**General description**	**Kit specifications**	**Cost/sample (USD)** ^**d**^
Qiagen RNeasy® Plus Mini (#74134)	Manual Operation^a^; Guanidine-isothiocyanate lysis, Silica membrane-based RNA purification, Non-enzymatic DNA elimination	Tissue Mass: ≤30 mg	$6.02
		Minimum Elution Volume: 30 μL^c^	
Qiagen RNeasy® Plus Universal Mini (#73404)	Manual Operation^a^; Phenol-guanidine lysis, Silica membrane-based RNA purification, Non-enzymatic DNA elimination	Tissue Mass: ≤50 mg	$6.86
		Minimum Elution Volume: 30 μL^c^	
Promega Maxwell® 16 LEV simplyRNA Purification (#AS1280)	Automated Operation; Guanidine-thiocyanate lysis, Paramagnetic bead-based RNA purification, Enzymatic DNA elimination	Tissue Mass: ≤50 mg	$5.25
		Minimum Elution Volume: 30 μL^c^	
Ambion MagMAX™-96 Total RNA Isolation (#AM1830)	Automated Operation^b^; Guanidinium thiocyanate-based lysis, Magnetic bead-based RNA purification, Enzymatic DNA elimination	Tissue Mass: ≤5 mg	$3.19
		Minimum Elution Volume: 20 μL	
Promega SimplyRNA HT System (Beta test kit^e^)	Automated Operation; Guanidine-thiocyanate lysis, Paramagnetic bead-based RNA purification, Enzymatic DNA elimination	Tissue Mass: 5–300 mg	$3.50-5.25
		Minimum Elution Volume: 30 μL	

^a^This kit can be automated on the Qiagen QIAcube robotic workstation.

^b^This kit can be used manually.

^c^For the Plus Mini, Plus Universal and Maxwell® kits, an elution volume of 30 μL was used for RNA extracted from blood, embryos and larvae, while an elution volume of 50 μL was used for RNA isolated from spleens and kidneys. Elution volumes for all tissues extracted via the MagMAX™ and SimplyRNA HT kits were 55 and 50 μL, respectively.

^d^List price in June 2013; Prices are for the kits only and do not include the costs of equipment and reagents not supplied with the kit.

^e^Beta test kit of a new 96-well format high throughput, RNA purification system. Final specifications in development.

### Tissue samples

All animal procedures in this study were approved by Miami University’s Institutional Animal Care and Use Committee. Fish were obtained from Miami University’s fathead minnow colony. Blood, kidneys and spleens were obtained from 20 sexually mature adult male fathead minnows following euthanasia via a lethal dose of MS-222. To obtain blood, the caudal peduncle was severed, blood was collected in a heparinized microhematocrit tube, transferred to 1.5 mL microcentrifuge tube and centrifuged to separate plasma, which was subsequently removed and discarded. Embryos (~72-84 h post fertilization, n = 20) and whole larvae (~168 h post hatch, n =20) were also sampled for analysis. All tissues were weighed immediately upon collection and masses are reported in Table 2. Tissues were flash frozen in liquid nitrogen and stored at −80°C until RNA isolation.

**Table 2:** **The average (± standard deviation) masses of tissues subject to RNA extractions via each kit**

	**Blood**	**Spleen**	**Kidney**	**Embryo**	**Larvae**
Plus Mini	19.5 (4.0)	5.3 (2.9)	20.3 (8.0)	1.1 (0.04)	6.4 (0.6)
Plus Universal	14.8 (2.8)	3.5 (1.2)	14.0 (4.6)	1.1 (0.1)	5.9 (0.8)
Maxwell®	20.0 (7.6)	3.7 (1.7)	13.4 (6.2)	1.1 (0.03)	6.7 (1.1)
MagMAX™	4.0 (0.7)^a^	1.7 (0.9)	3.8 (1.8)^a^	1.1 (0.1)	6.5 (0.5)
SimplyRNA HT	11.3 (3.8)	4.9 (3.4)	14.3 (7.9)	1.2 (0.1)	5.3 (1.9)

^a^Tissues processed using the MagMAX™ kit were trimmed to achieve manufacturer suggested tissues masses ≤5 mg.

### RNA extraction

For each of the kits tested, tissues were homogenized in the manufacturer supplied homogenization/lysis buffer with a Misonix Microson XL-2000 Ultrasonic Cell Disruptor (QSonica, LLC., Newtown, CT). The specifications of the Plus Mini, Plus Universal, Maxwell® and SimplyRNA HT kits were such that tissues did not require trimming prior to homogenization to achieve manufacturer suggested tissue amounts. However, the mass of blood, kidney and larvae samples designated for processing using the MagMAX™ kit were often greater than the recommended tissue amount of 5 mg. To account for this, the blood and kidneys samples were trimmed (while frozen) to achieve masses ≤5 mg. Given that the cellular composition of larvae is far from homogeneous and that the mass of larvae was just above the recommended mass of 5 mg, larvae were left intact. For each tissue type, statistical analysis was conducted to determine if there were differences in the masses of tissues designated for extraction via each of the kits. Tissues that were trimmed for use with the MagMAX™ kit were excluded from this analysis. For each tissue type, statistical analysis confirmed that there were no significant differences in the amount of starting material processed by each kit (ANOVA, *p* >0.13 in all cases; Table 2).

RNA extractions using the Plus Mini and Plus Universal kits were performed manually. Extractions using the Maxwell® kit were performed on a Promega’s robotics platform, the Maxwell® 16 Research System, while those using the MagMAX™ and SimplyRNA HT kits were performed on a Beckman Coulter BiomekFX robot equipped with both an AP96 and a SPAN8 pod. The difference between the two robotics platforms is that the BiomekFX liquid handling robot can transfer liquids from one location to another, whereas the Maxwell® 16 transfers the paramagnetic beads – not the liquid. Manufacturer protocols for the Plus Mini, Plus Universal were followed without exception. Protocols for the Maxwell® kit were followed with one exception - all samples were homogenized in 210 μL of homogenization solution, rather than the recommended 200 μL, to ensure adequate volumes of homogenate for subsequent procedures. The method for Simply RNA HT kit was optimized for the deck configuration utilized in Miami University’s Center for Bioinformatics and Functional Genomics, and the method for the MagMAX™ kit was re-written from the provided BiomekNX protocol to be compatible with the BiomekFX and BiomekFX Software 3.3.14 (see Additional file 1). With the MagMAX™ kit, we experienced problems with the high viscosity of the magnetic bead mix included with the kit. To overcome this problem, the bead mix was diluted with additional lysis buffer (2.2 parts bead mix: 1 part lysis buffer) to ensure accurate pipetting by the BiomekFX. To account for the dilution of the magnetic beads, the amount of bead mixture utilized was increased from 20 μl to 30 μl per extraction to maintain manufacturer recommended amounts of magnetic particles in each extraction well.

### Determination of RNA quantity and quality

Immediately following extraction, the total RNA concentration and A_260_:A_280_ ratio of each sample was determined via NanoDrop 2000 (Thermo Scientific). Total RNA concentrations were converted to total amounts of RNA extracted (μg) to account for differences in the required elution volumes (see Table 1) of each kit. In addition, because the amount of starting tissue can impact recovery, the RNA recovered per mass of input tissue (μg RNA/mg tissue) was also determined. RNA integrity numbers (RIN) were determined using the RNA 6000 NanoKit for the Bioanalyzer 2100 (Agilent Technologies, Santa Clara, CA) per manufacturer protocol. The threshold cycle (Ct) values were determined for each of the selected samples with and without the additional DNase treatment and in the corresponding RT(−) samples to determine the effectiveness of gDNA removal by each of the kits.

### Evaluation of the effectiveness of gDNA elimination

Genomic DNA contamination was evaluated by conducting qPCR reactions on RT(−) samples [13]. Because the RT(−) samples have not undergone reverse transcription, they should contain only RNA. Hence, RT(−) samples that are free of gDNA should not generate a fluorescent signal during qPCR reactions. Here, one sample per tissue per kit was randomly selected so that the ability of each kit to remove gDNA could be evaluated. Total RNA samples were considered gDNA free if the Ct values of the corresponding RT (−) samples were ≥35, as these values are typically considered noise [21,22]. The difference between the Ct values of the RT (−) and RT (+) samples was also determined. Differences ≥5 cycles are typically taken to indicate that gDNA contamination will not adversely impact qPCR results, as differences of this magnitude indicate that <3% of the fluorescent signal generated during the qPCR reaction results from the presence of gDNA [23].

Each of the RNA samples selected for this analysis was subjected to a second DNase treatment via Invitrogen’s DNA-*free* kit (Life Technologies, Grand Island, NY) per manufacturer protocol to determine whether additional DNase treatment was needed to supplement the DNA removal procedures utilized by each of the kits. Because the quantity of RNA extracted from embryos was less than the amount required for use with the DNA-*free* kit, RNA samples from embryos were pooled prior to treatment and only pooled samples were used in subsequent analysis.

### Quantitative PCR

First-strand cDNA was synthesized for each of the RNA samples selected for the additional DNase treatment, as well as the corresponding total RNA samples prior to the additional treatment. The iScript cDNA Synthesis Kit (Bio-Rad Inc., Hercules, CA) was utilized for first-strand cDNA synthesis. Briefly, reactions were carried out via a PTC-100 thermal cycler (MJ Research, Waltham, MA; 25°C for 5 min, 42°C for 30 min, 85°C for 5 min) with 0.1 μg total RNA in 7.5 μl nuclease-free water, 2 μl iScript reaction mix and 0.5 μl iScript reverse transcriptase. The resulting cDNA was diluted with nuclease-free water to a final volume of 40 μL.

Following first-strand synthesis, the cDNA samples (and corresponding total RNA samples) were shipped on dry ice to Texas Christian University (Fort Worth, TX) where qPCR was utilized to quantify the expression of glutathione S-transferase (GST). All qPCR reactions were performed in triplicate using a CFX Connect real-time PCR detection system managed by CFX Manager Software version 3.0 (Bio-Rad). Each 10 μl reaction contained 0.4 μL cDNA, 5 μL iQ SYBR-Green supermix (Bio-Rad) and 300 nM of forward and reverse primer. The GST primers were designed using CLC Genomics Workbench 6.8.4 (CLC bio, Cambridge, MA), synthesized by Integrated DNA Technologies (Coralville, IA), and had the following sequences: forward 5′-GGGCATTGGTGATCTAAC-3′ and reverse 5′-TTTTCAAACACAGGGAGG-3′. All reactions were carried out in white shell/clear well PCR plates (Bio-Rad) and the thermal cycling program consisted of an activation step (95°C, 3 min) followed by 40 cycles of denaturing (95°C, 10 sec) and annealing (50°C, 45 sec). The threshold cycle (Ct) values were determined for each of the selected samples with and without the additional DNase treatment and in corresponding RT (−) samples.

### Data analysis

For each tissue type, significant differences in the quantity (*i.e.*, total RNA amount and mass-adjusted total RNA amount) and quality (*i.e.*, A_260_:A_280_ ratio and RIN value) of RNA extracted using each kit were evaluated via one-way analysis of variance (ANOVA, JMP Pro 10.0.0, SAS Institute) followed by Tukey’s multiple comparisons tests. In cases where the assumption of homogeneity of variances was not met, a Welch ANOVA followed by Wilcoxon multiple comparisons tests was conducted. Kit precision was evaluated by testing for unequal variances (Levene’s test) between each of the RNA quantity and quality parameters measured and through the calculation of coefficients of variation (%CV). Statistical significance was declared at *p* <0.05.

The ability of each kit to produce RNA meeting quality and quantity criteria likely to be sufficient for qPCR, microarray and NGS analysis was also assessed. Several previous studies have established the minimum quantity and quality of RNA designated for use in downstream gene expression analysis [1,14,15,17,19,20]. To determine whether the RNA isolated via the kits evaluated in this study was sufficient for qPCR, microarray and NGS analysis, we determined whether the total amount of RNA recovered, the A260:A280 ratios and the RIN values of each sample met the minimum quality and quantity criteria shown in Table 3.

**Table 3:** **The minimum quantity and quality criteria for RNA used to evaluate the suitability of RNA extracted via each kit for analysis via real-time quantitative (qPCR), microarray or next generation sequencing (NGS) technologies**

	**Total RNA quantity**	**A**_**260**_ **:A**_**280**_ **ratios**	**RNA integrity number (RIN)**
qPCR	≥ 1 μg	≥ 1.8	≥ 5
Microarray	≥ 5 μg	≥ 1.8	≥ 7
NGS	≥ 1 μg	≥ 1.8	≥ 7

## Results and discussion

**Figure 2:**
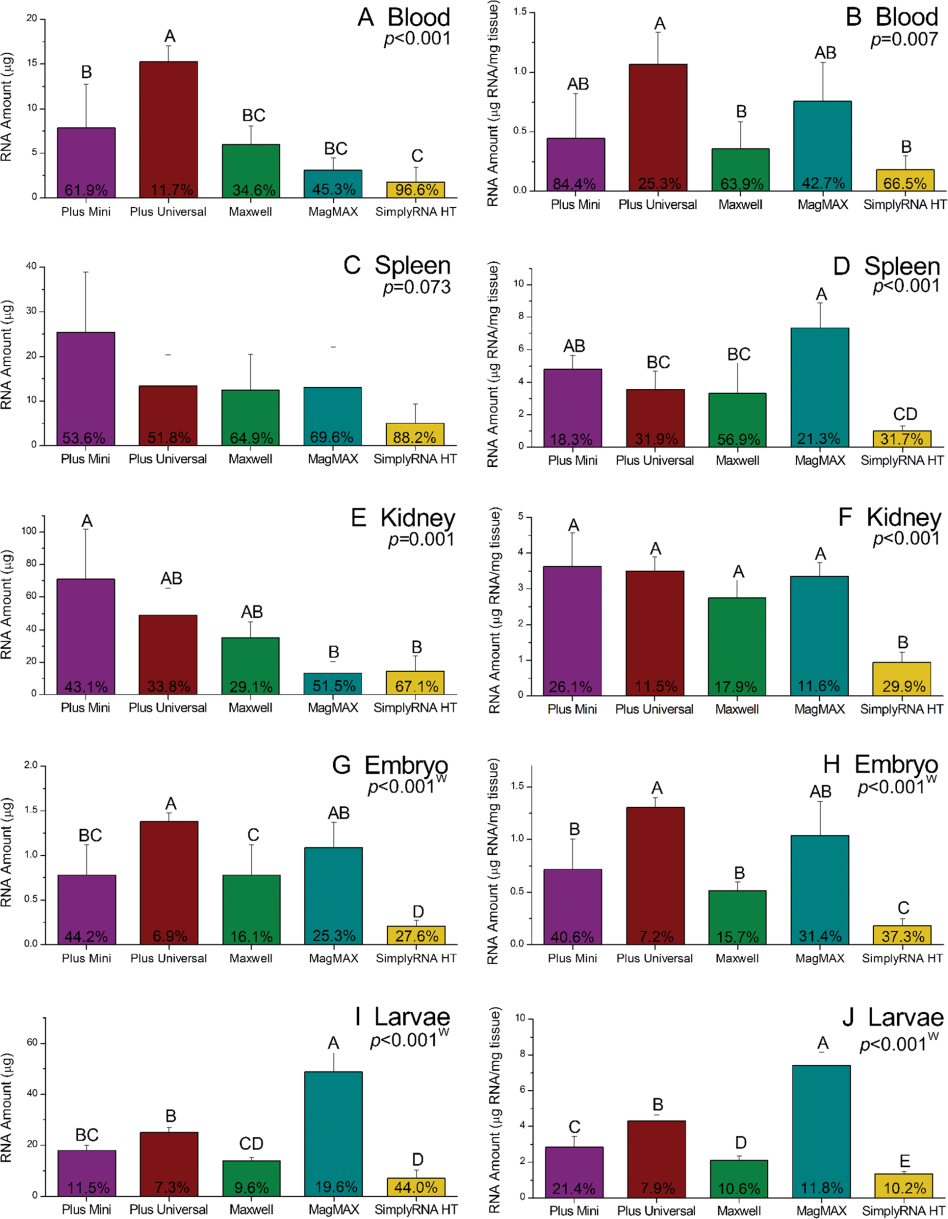
**The quantity of RNA extracted from fathead minnow tissues using five commercially available RNA extraction kits.** The RNA amounts shown in panels on the left **(A, C, E, G** **and** **I)** reflect the total amount of RNA recovered (ng), while the RNA amounts reported in panels on the right **(B, D, F, H** **and** **J)** indicate the amount (ng) of RNA recovered per mass (mg) of tissue. The reported p-values were obtained by one-way ANOVA, except for those denoted by a “W”. The “W” indicates that significant differences in variance were detected and that analysis was conducted via a Welch ANOVA. Error bars indicate standard deviation. Different letters above bars indicate significant differences in the measured RNA quantity parameter between samples processed by each of the kits. Percentages reported in each bar are indicative of the coefficient of variance.

**Figure 3:**
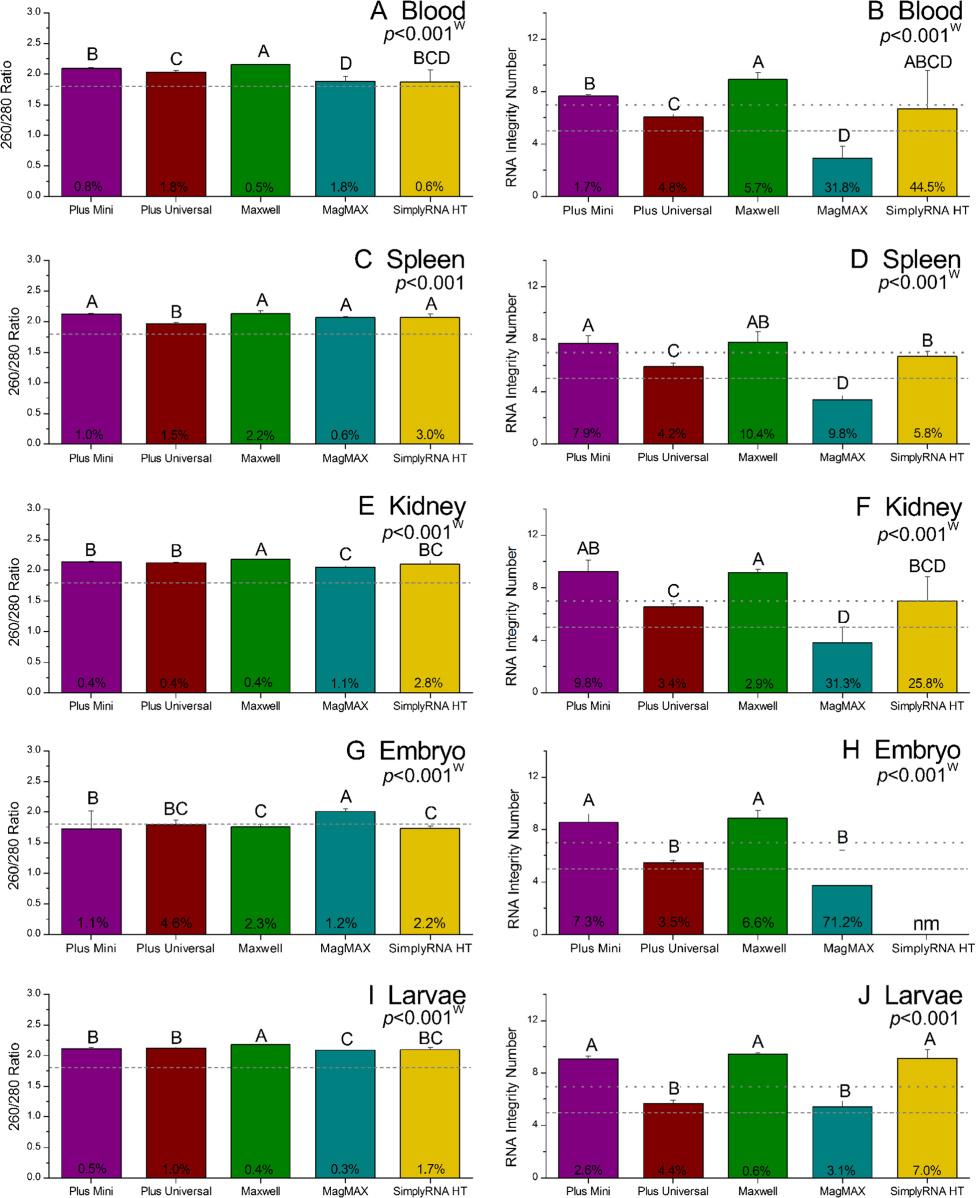
**The quality of RNA extracted from fathead minnow tissues using five commercially available RNA isolation kits.** The panels on the left **(A, C, E, G** **and** **I)** show the 260/280 ratios of RNA extracted using each kit; the dashed lines on each panel represents a 260/280 ratio of 1.8 (the value above which RNA is considered to be “pure”). The panels on the right **(B, D, F, H** **and** **J)** show the RNA integrity number (RIN); the dashed line represents a RIN of 5 (the minimum acceptable RIN for qPCR analysis), while the dotted line shows a RIN of 7 (the minimum recommended RIN for microarray and next generation sequencing analyses). Reported p-values were obtained by one-way ANOVA, except for those denoted by a “W”. The “W” indicates that significant differences in variance were detected and that analysis was conducted via a Welch ANOVA. Error bars indicate standard deviation. Different letters above bars indicate significant differences in the measured RNA quantity parameter between samples processed by each of the kits. Percentages reported in each bar are indicative of the coefficient of variance. RIN values were not measurable (indicated by “nm”) in RNA extracted from embryos using the SimplyRNA HT kit due to low RNA concentrations.

The performance of each RNA extraction kit was evaluated for each tissue type. Significant differences in the both the quantity and quality of RNA recovered by each were detected (Figures 2 and 3). However, we recognize that in several cases statistically significant differences did not translate to practical differences in RNA quantity or quality (*i.e.*, the statistical analysis was more sensitive than analytically relevant). For example, there were significant differences in amount of RNA recovered from blood, kidney and larvae by each kit (Figure 2, ANOVA, *p* <0.001). However, the average amount of total RNA recovered from these tissues (regardless of the kit used) exceeded the minimum amount considered sufficient for most downstream molecular applications, suggesting a lack of practical difference between the kits with regard to RNA yield from these tissue types. Likewise, there were instances where statistical differences in RNA quality were not necessarily indicative of practical differences in RNA quality. This was particularly true for A_260_:A_280_ ratios. For each tissue, there were significant differences in the A_260_:A_280_ ratios of RNA recovered by each of the kits (Figure 3, ANOVA/Welch ANOVA, *p* <0.03). However, for all but embryos, the average A_260_:A_280_ ratios of total RNA samples were ≥1.8 (Figure 3) indicating that each kit was capable of producing “sufficiently pure” RNA suitable for gene expression analysis by qPCR, microarray, or NGS. Furthermore, an analysis to determine the minimal detectable differences between the A_260_:A_280_ ratios of samples was conducted and this analysis revealed that in most cases, differences of <0.1 could be detected. Differences of this magnitude would not be considered relevant from an analytical perspective. As such, subsequent discussions of kit performance will focus primarily on practical differences, rather than statistical differences.

### Quantity and quality of RNA recovered via each of the kits

While some of our results are tissue-dependent, a few general trends are particularly noteworthy with regard to the quantity and quality of RNA recovered by each of the kits. First, for each tissue (except embryos), the amount of RNA recovered from each sample was ≥1 μg, regardless of the kit used indicating that each of the kits evaluated was capable of recovering sufficient amounts of total RNA for analysis via qPCR or NGS technologies (Figure 2, Table 3). Next, nearly all of the RNA samples had A_260_:A_280_ ratios ≥1.8 (Figure 3). Only embryos extracted via the Plus Universal, Maxwell® and SimplyRT HT kits and blood extracted via the Simply RT HT kit had A_260_:A_280_ ratios <1.8 and these less-than-optimal ratios were likely related to low concentrations (<15 ng/μL in all cases) of nucleic acid in these samples [24], rather than the ability of the kit to remove contaminants that absorb at 280 nm. As such, it appears that there were few practical differences between the kits with regard to the removal of contaminants that absorb at 280 nm.

**Figure 4:**
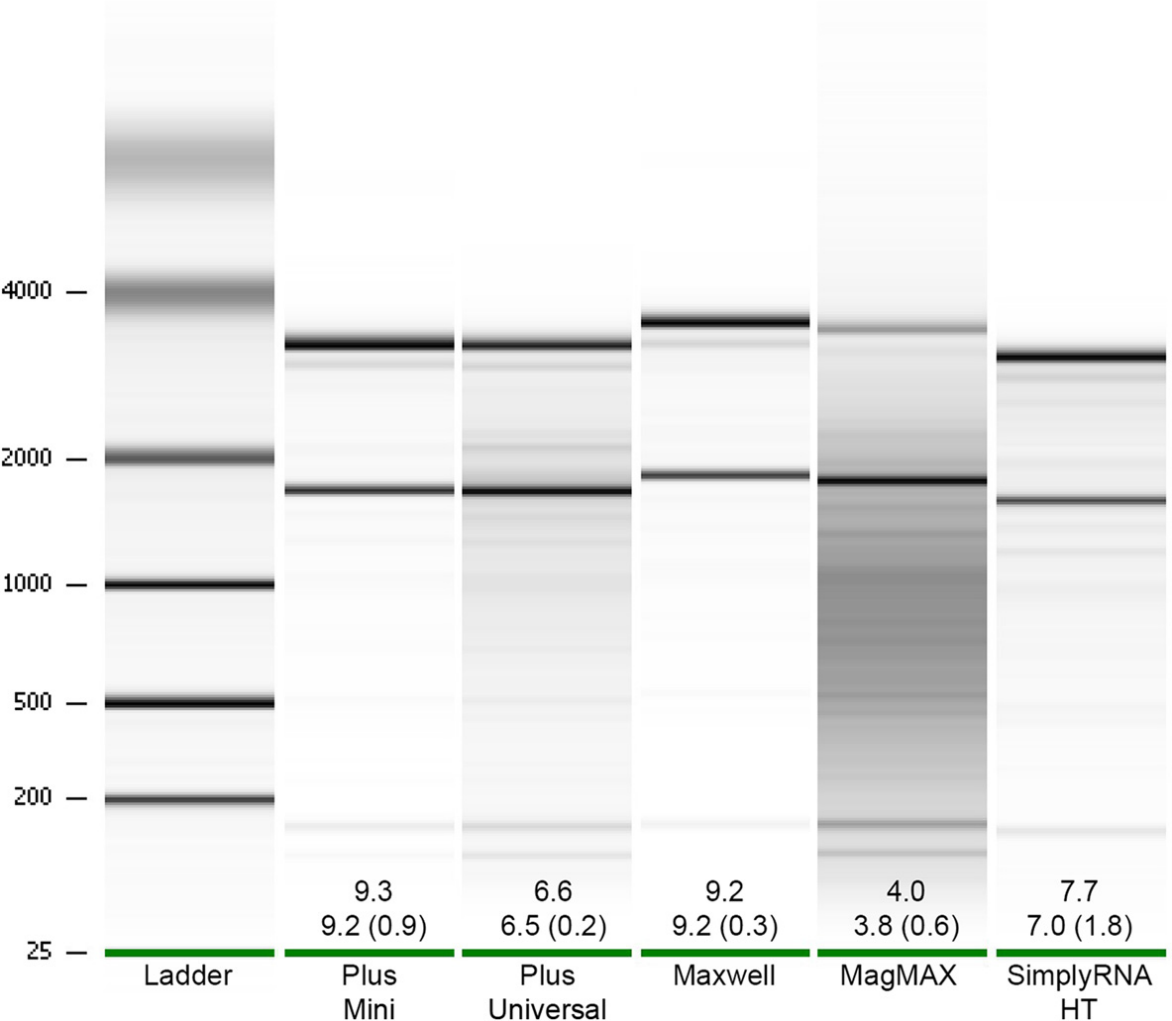
**A representative example of the gel images produced during RNA quality checks performed via the Bioanalyzer 2100.** The composite gel image was generated from the results of RNA quality assessments performed on RNA extracted from larvae samples. For each kit, the lane corresponding to the sample that best represented the “average” RNA yielded is included. The first number reported for each kit represents the RIN value of the sample shown, while the numbers below represent the mean RIN value (± standard deviation) for all larvae samples extracted via each kit. Note that the lanes shown in the image were not obtained from samples run on the same Nanochip resulting in the misalignment of the 18S and 28S bands between some of the lanes.

The most relevant differences in the RNA extracted via each kit were related to the ability of the kits to consistently extract intact total RNA. Figure 3 shows the average RIN values obtained for each tissue extracted via each of the kits, while Figure 4 provides a representative example of the gel images obtained for samples extracted via each of the kits. The Plus Mini and Maxwell® kits yielded the most intact (*i.e.,* least degraded) total RNA as evidenced by the fact that the majority of samples extracted via these two kits (regardless of tissue type) had average RIN values ≥7 (Figure 3). The performance of the Plus Universal and SimplyRNA HT kits was similar with regard to the integrity of the RNA yielded. For most tissues, these two kits yielded RNA with average RIN values ≥5 (but often <7) indicating RNA with a moderate amount of degradation. The RNA extracted via the MagMAX™ kit typically was found to be highly degraded in most cases, as average RIN values were <5 for each of the tissue types except for larvae. These findings demonstrate differences in the ability of each kit to extract intact RNA and suggest that some kits may be better suited for specific downstream applications than others.

Finally, a majority of the samples tested negative for gDNA contamination (Table 4), with the exception of samples isolated via the MagMAX™ kit, suggesting that most of the kits evaluated here offer near complete removal of gDNA from tissue samples. In cases where samples tested positive for gDNA, an additional post-extraction DNase I treatment was capable of eliminating this contamination suggesting that the performance of kits with less than optimal gDNA removal can be enhanced by an additional DNA removal procedure.

**Table 4:** **Ability of each kit to effectively remove DNA from small tissue samples**

	**Pre-DNase**			**Post-DNase**		
	**no RT**	**RT**	**Difference**	**no RT**	**RT**	**Difference**
*Plus Mini*						
Blood	33.65	31.71	1.94	> 40*	32.23	7.77^†^
Spleen	39.84*	28.62	11.22^†^	39.84*	29.59	10.25^†^
Kidney	38.06*	19.35	18.71^†^	> 40*	23.41	16.59^†^
Embryo	> 40*	27.82	12.18^†^	> 40*	29.46	10.54^†^
Larvae	> 40*	26.26	13.74^†^	> 40*	27.86	12.14^†^
*Plus Universal*						
Blood	33.27	32.87	0.40	> 40*	34.07	5.93^†^
Spleen	34.77	28.90	5.87^†^	39.92*	29.14	10.78^†^
Kidney	36.50*	21.54	14.96^†^	> 40*	22.18	17.82^†^
Embryo	39.79*	29.23	10.56^†^	> 40*	31.17	8.83^†^
Larvae	> 40*	20.74	19.26^†^	> 40*	28.79	11.21^†^
*Maxwell*®						
Blood	> 40*	34.19	5.81^†^	> 40*	34.84	5.16^†^
Spleen	> 40*	29.38	10.62^†^	> 40*	30.25	9.75^†^
Kidney	> 40*	20.52	19.48^†^	> 40*	20.67	19.33^†^
Embryo	37.09*	26.41	10.68^†^	> 40*	25.86	14.14^†^
Larvae	> 40*	26.46	13.54^†^	> 40*	27.55	12.45^†^
*MagMAX* ^*™*^						
Blood	29.79	28.11	1.68	39.02*	32.65	6.37^†^
Spleen	> 40*	29.93	10.07^†^	38.04*	30.36	7.68^†^
Kidney	31.83	21.89	9.94^†^	39.74*	21.51	18.23^†^
Embryo	36.34*	22.57	13.77^†^	> 40*	22.31	17.69^†^
Larvae	33.75	27.54	6.21^†^	> 40*	28.17	11.83^†^
*SimplyRNA HT*						
Blood	> 40*	31.06	8.94^†^	> 40*	31.55	8.45^†^
Spleen	> 40*	28.11	11.89^†^	> 40*	27.9	12.10^†^
Kidney	> 40*	21.54	18.46^†^	> 40*	21.94	18.06^†^
Embryo^a^	-	-	-	-	-	-
Larvae	> 40*	26.22	13.78^†^	> 40*	26.00	14.00^†^

The threshold cycle (Ct) values of samples subjected to the kit DNase treatment only (pre-DNase) or to an additional DNase treatment, as well as the corresponding “no RT” samples (see Methods for details). *represents no RT samples with Ct values ≥35 and thus, considered gDNA free. ^†^indicates samples for which difference between the Ct values of the no RT and RT samples was ≥5, an indicator of sufficient gDNA removal for qPCR gene expression analysis.

^a^The quantities of RNA isolated from embryos using the SimplyRNA HT kit were less than the amount necessary for the first-strand synthesis protocol used in this study; therefore, qPCR reactions could not be performed with these samples.

### Tissue specific considerations

#### Blood

In the current study, RNA was extracted from whole blood samples and thus, the reported total RNA yields include the amount of globin RNA in the sample. Blood samples are frequently subject to globin reduction procedures prior to RNA extraction; therefore, it is reasonable to suspect that the amount of total RNA recovered from blood samples would be lower than those reported if a globin reduction step had been included in the RNA extraction procedure. A study by Mastrokolias et al. [25] found that globin reduction procedures reduced the total RNA content of samples by 5-9%. Furthermore, Mastrokolias et al. [25] also found that globin reduction led to a slight decrease in RIN values, as non-globin reduced samples had average RIN values of approximately 7.7, while those subject to globin reduction had average RIN values of 7.3. Given these results, it is likely that globin reduction would not only reduce the quantity total RNA obtained from blood samples, but also the quality of the recovered RNA.

#### Spleens

Spleens are frequently considered a “difficult” tissue due to their relatively high abundance of nucleases [26] which makes the RNA present in spleen tissue more prone to degradation. In the current study, we found that the RNA from spleen samples tended to be less intact than that from kidney, embryo or larvae samples. For example, kidney and larvae samples extracted via the Plus Mini and Maxwell® kits had average RIN values >9. In contrast, spleen samples extracted with these kits had average RIN values of 7.7 and 7.8, respectively. Though the ultimate cause of these seemingly tissue-specific differences in the integrity of RNA recovered from is unknown, it is possible that the inherent nuclease content spleen indeed made the RNA therein more subject degradation than the RNA in kidney and larvae samples.

#### Embryos

For single embryos, the amount of RNA recovered (regardless of the kit utilized) was relatively low in comparison to other tissues and ranged from an average of 0.21 to 1.38 μg. The Plus Universal and MagMAX™ kits were capable of extracting ≥1 μg total RNA, an amount often suitable for qPCR and NGS analysis. However, there were differences in the quality of RNA obtained using these kits. Specifically, the Plus Universal kit produced moderately degraded RNA (average RIN =5.5), while the MagMAX™ kit yielded highly degraded RNA (average RIN =3.8). Assuming that NGS analysis requires RNA with RIN values ≥7, these results suggest that neither the Plus Universal nor the MagMAX™ kit produces RNA from single embryos that is suitable for NGS experiments. Given that moderately degraded RNA can often produce successful qPCR results, it is possible that embryonic RNA extracted via the Plus Universal kit may be acceptable for qPCR analysis. The suitability of the Plus Mini, Maxwell® and SimplyRNA HT kits for embryonic RNA extractions appears to limited by the quantity of RNA yielded (<1 μg for each kit), not the quality of RNA yielded (RNA ≥7 for RNA extracted from the Plus Mini and Maxwell® kits). Given this, coupled with the fact that extractions were performed on single embryos, it is possible that the performance of these kits could be improved by pooling embryos prior to extraction. Fromm [11] found that not only the quantity, but also the quality of RNA isolated from pooled *Gyrodactylus salaris* samples (consisting of either 10 or 100 individuals) was greater than that from single *G. salaris*, providing evidence that sample pooling can be utilized to optimize RNA isolation.

### The extraction of RNA for downstream gene expression analysis

When determining whether an RNA sample is acceptable for downstream gene expression analysis, the quantity and quality of RNA are generally the primary factors considered. In order to evaluate the performance of each kit in its ability to produce RNA suitable for qPCR, NGS and microarray analysis, we established minimum RNA quantity and quality criteria specific to each type of analysis (Table 3). With regard to RNA quantity, the total RNA amount required for first-strand cDNA synthesis and thus, qPCR and NGS is typically in the picogram (pg) to nanogram (ng) range [14]. In contrast, the labeling protocols for microarray analysis frequently require ≥5 μg of total RNA [15]. Like RNA quantity, the RNA quality required for gene expression analysis is dependent upon the method used to quantify expression. To evaluate the ability of each kit to produce RNA of suitable quality for qPCR, NGS and microarray experiments, we assumed that samples with RIN values ≥5 met the minimum quality criteria for analysis via qPCR and that those with values ≥7 met the minimum quality criteria for analysis via microarray or NGS analysis (Table 3). Though the assumptions were made based upon several accounts in the literature [1-3,7,19,20], it would be naïve to assume that the RIN value criteria set here is universally applicable and thus, we recommend that individual labs establish RIN value criteria more specific to their experimental conditions (*i.e.,* sample type, quantitation method, amplicon length, etc.).

#### qPCR analysis

Embryos aside, the Plus Universal and MagMAX™ kits generally yielded higher amounts of RNA per mg of tissue than the other kits, while the SimplyRNA HT kit consistently yielded the lowest amount of RNA. Despite significant differences in the quantity of RNA recovered by each kit, the amount of RNA recovered from each tissue sample was ≥1 μg, independent of the kit used - with the exception of blood extracted via the SimplyRNA HT kit (average yield of 1.74 ± 1.7 μg). Our results indicate that each of the kits tested is likely to extract quantities of total RNA sufficient for most qPCR. A closer examination of the quality of RNA extracted using each of these kits, revealed that the Plus Mini, Plus Universal and Maxwell® kits recovered RNA with A_260_:A_280_ ratios ≥1.8 and RIN ≥5 from blood, spleen, kidney and larvae indicating that RNA extracted from these tissues via these kits is acceptable for qPCR. Likewise, the SimplyRNA HT kit extracted RNA meeting the quantity and quality criteria for qPCR analysis from spleen, kidney and larvae; though in our hands, the ability of this kit to extract suitable RNA from blood samples was less consistent. The quality of RNA extracted from the larval samples via the MagMAX™ kit met the minimum quality criteria for qPCR (A_260_:A_280_ ratios >1.8 and RIN value >5). However, none of the RNA samples extracted from blood, spleens and kidneys met these criteria suggesting that the MagMAX™ kit may be limited in its ability to extract intact RNA.

#### Microarray and NGS analysis

Whereas each of the kits except the MagMAX™, were capable of isolating RNA meeting the criteria for qPCR analysis, only the Plus Mini and Maxwell® kits were consistent in their ability to isolate RNA meeting the assumed criteria for microarray analysis. Nearly all of the RNA extracted from blood, spleen, kidney and larvae samples by these two kits contained RNA of acceptable quantity (1 and 5 μg total RNA for NGS and microarray analysis, respectively) and quality (RIN values ≥7). Likewise, the SimplyRNA HT had similar performance characteristics for kidney and larvae samples with the majority of samples fulfilling the criteria for microarray and NGS analysis. However, this kit was less consistent in its ability to extract RNA from blood and spleen as the majority of the blood and spleen RNA samples extracted with this kit did not meet the minimum quantity requirements for microarray analysis. Furthermore, the majority of spleen samples isolated with this kit did not fulfill the quality requirements for microarray or NGS analysis. This indicates that the SimplyRNA HT may not be sufficient for extracting blood and spleen samples for use in microarray analysis; although, it is possible that increasing the amount of starting material could potentially overcome the low yield observed for the SimplyRNA HT blood and spleen samples. It should also be noted that these inconsistencies in the quantity of RNA recovered via the SimplyRNA HT kit may be an artifact of this kit being in the beta test stage (at time of assay) and not being quite fully optimized for our BiomekFX platform – to our knowledge, we were among the first groups outside of Promega to evaluate this kit. The Plus Universal kit consistently produced RNA samples of sufficient quantity; however, none of the samples extracted with this kit had RIN values ≥7 suggesting that the RNA recovered via this kit is subject to fragmentation and may be less than optimal for microarray and NGS applications.

### Effectiveness of kit DNA elimination

Expression analysis of samples prior to the additional DNase treatment showed the RT (−) reactions conducted with samples extracted via the Maxwell® and SimplyRNA HT kits were gDNA free (as indicated by Ct values >35, Table 4) suggesting that the DNA removal procedures utilized by these kits (*i.e.,* DNase I treatment) is effective for the tissue types evaluated here. Similarly, the Plus Mini and Plus Universal kits offered near complete removal of DNA from most of the samples indicating that the DNA removal procedure utilized by these kits (*i.e.,* non-enzymatic removal) is sufficient for most tissues. In contrast, the majority of the MagMAX™ samples evaluated for gDNA contamination tested positive, indicating that this kit is inconsistent in its ability to effectively remove DNA. It should be noted that an additional DNAse I treatment was effective in removing DNA from all of the samples that tested positive for gDNA contamination. Thus, the performance of kits with seemingly ineffective gDNA removal capabilities can be easily augmented via additional DNA removal procedures.

Some downstream molecular procedures do require samples that are almost completely free of gDNA contamination (*i.e.,* NGS analysis in which a single contaminating gDNA strand can be detected) [27]; however, not all molecular applications require this degree of gDNA removal. For example, in qPCR reactions, slight gDNA contamination is acceptable if the presence of the gDNA does not account for more than 3% of the fluorescent signal (indicated by differences in the Ct values of RT(−) and RT(+) samples of >5) [23]. A comparison of RT(−) and RT(+) values revealed that for many of the samples that tested positive for gDNA contamination, namely the spleen sample from Plus Universal kit and the kidney and larvae samples from the MagMAX™ kit, the level of gDNA contamination was unlikely to impact the results of qPCR as the difference between the RT(−) and RT(+) Ct values was >5 in all cases. However, for blood samples extracted by the Plus Mini, Plus Universal, and MagMAX™ kits, the difference between the Ct values was well under 5 indicating that the presence of gDNA in these samples will negatively affect gene expression data obtained via qPCR. As such, an additional DNase treatment is likely required for blood samples extracted via these kits regardless of the downstream molecular application to be used.

### Kit precision

A consideration of kit precision is important in evaluating the overall performance of each kit. For each of the RNA quantity and quality parameters measured, differences in variance were evaluated. With regard to variation in the amount of total RNA recovered by each kit, no significant differences in variance were detected, with the exception of embryos, indicating that the consistencies of each kit with regard to the amount of RNA extracted were similar. For embryos, significant differences in variances of the amount of total RNA isolated were detected (Levene’s test, *p* =0.004). An examination of the %CV for the amount of RNA recovered by each kit showed that the Plus Universal Kit and Maxwell® kits were the most consistent in their ability to isolate RNA (%CV =6.9 and 16.1, respectively). In contrast, the Plus Mini, MagMAX™ and SimplyRNA HT kits were less consistent (%CV >25 in each case). Given that many of the embryo RNA samples contained amounts of RNA near the minimum amount required for qPCR and NGS analysis (*i.e.,* 1 μg), the precision of these kits is of particular importance. This is borne out when comparing the Plus Mini and Plus Universal kits, as the average amount of embryonic RNA isolated by each of these kits was >1 μg (Figure 2). However, a closer examination showed that 100% of the embryo samples extracted via the Plus Universal kit, whereas only 25% of the samples isolated by the Plus Mini kit contained, contained >1 μg of total RNA.

With regard to RNA quality, there were differences in the variance associated with the A_260_:A_280_ ratios for blood, kidney, embryo and larval RNA samples isolated via the five kits evaluated (Levene’s test, *p* <0.03 in each case). Despite these differences in variance, the %CV associated with the A_260_:A_280_ ratios for each of these tissues extracted by each of the kits was ≤3% in all but one case, suggesting acceptable consistency in the ability of each of the kits to isolate “pure” RNA. The one exception to this appears to be the SimplyRNA HT kit, as the %CV associated with the A_260_:A_280_ ratios for blood RNA samples was 10.6%. This in combination with the fact that half of the blood samples extracted via this kit had A_260_:A_280_ ratios <1.8 indicates that the SimplyRNA HT kit lacked precision in isolating “pure” RNA from blood samples. However, it is plausible that the higher variance in A_260_:A_280_ ratios for blood RNA samples isolated with the SimplyRNA HT kit is related to the relatively low amount of RNA yielded from these samples, as low A_260_:A_280_ ratios can result from low concentrations of RNA [24].

Significant differences in the variance associated with RIN values of RNA obtained via the kits were noted for each tissue type (Levene’s test, *p* <0.04 in each case), except for larvae. This result suggests that the kits differed with regard to their consistency in extracting intact RNA. An examination of %CVs revealed that the Plus Mini, Plus Universal, and Maxwell® kits offered the highest degree of consistency regardless of tissue type, as the %CVs were < ~10. The MagMAX™ and SimplyRNA HT kits offered suitable consistency for spleen and larvae samples (%CV <10); however, these kits lacked consistency for blood, kidney and embryo samples (%CV >25 in each case). Overall, these findings suggest that the Plus Mini, Plus Universal, and Maxwell® kits offer more precision in isolating intact RNA than the MagMAX™ and SimplyRNA HT kits. The reduced precision associated with the MagMAX™ and SimplyRNA HT kits may be related to the performance of the kits themselves. Alternatively, it is possible that the observed lack of consistency was related to the fact that both of these kits were utilized in conjunction with the BiomekFX robot. Thus, the possibility that the performance of the robot introduced an additional source of variation cannot be excluded.

## Conclusion

In summary, we have compared the ability of five commercially available RNA isolation kits to yield sufficient amounts of high-quality RNA from small tissue samples for downstream gene expression analysis. For low-throughput RNA isolation, we found the performance of the Plus Mini and Maxwell® kits to be similar, as both were consistent in their ability to extract relatively large quantities of intact RNA. Given the similar performance of these kits, considerations of cost and technician time are warranted. With regard to cost, the per sample costs associated with the Maxwell® kit is a bit lower than that for the Plus Mini; however, the Maxwell® kit must be used in conjunction with a Maxwell® 16 Instrument (list price of ~ $24,000). The amount of time required for RNA extractions via these kits was not specifically measured in this study. However, Qiagen reports that the amount of time required for the Plus Mini kit is 25 m [28] and we estimate that Maxwell® kit requires ~75 m (~20 m ‘hands-on’ time and 55 m processing time in the Maxwell® instrument). As such, the Plus Mini kit appears to more cost- and time-effective than the Maxwell® kit, though the automation of the Maxwell® kit is likely to reduce the possibility of technician error. For high-throughput RNA extraction, we found that for most tissues, the SimplyRNA HT kit was able to isolate RNA that meets the quantity and quality criteria for downstream analysis via qPCR, microarray and NGS technologies. However, this kit offered less precision than any of the low-throughput kits with regard to the quality of RNA extracted. At the time of this study, the SimplyRNA HT system was at the end of beta testing, and future refinements to the kit may increase yield quantity, quality and precision. It should also be noted that RNA extractions using the Plus Mini and Plus Universal kits can be conducted with the QIACube robotics platform. Additional studies aimed at evaluating the performance of these kits on the QIACube platform are warranted, as are inter-laboratory studies aimed at cross-validating the results presented here.

## Additional file

Additional file 1:
**BiomekFX protocols for used for RNA extractions using the MagMAX™ and simplyRNA HT isolation kits.**
